# Why Uptake of Blended Internet-Based Interventions for Depression Is Challenging: A Qualitative Study on Therapists’ Perspectives

**DOI:** 10.3390/jcm9010091

**Published:** 2019-12-30

**Authors:** Mayke Mol, Claire van Genugten, Els Dozeman, Digna J. F. van Schaik, Stasja Draisma, Heleen Riper, Jan H. Smit

**Affiliations:** 1 Department of Research and Innovation, GGZ inGeest, Specialized Mental Health Care, 1081 Amsterdam, The Netherlands; c.genugten@ggzingeest.nl (C.v.G.); e.dozeman@ggzingeest.nl (E.D.); a.schaik@ggzingeest.nl (D.J.F.v.S.); s.draisma@ggzingeest.nl (S.D.); h.riper@vu.nl (H.R.); jh.smit@ggzingeest.nl (J.H.S.); 2 Department of Psychiatry, Amsterdam Public Health Research Institute, VU University Medical Center, 1081 Amsterdam, The Netherlands; 3 Department of Clinical, Neuro and Developmental Psychology, Clinical Psychology Section, Vrije Universiteit Amsterdam and the Amsterdam Public Health Research Institute, 1081 Amsterdam, The Netherlands; 4 Telepsychiatry and E-Mental Health, Department of Clinical Research, University of Southern Denmark, 5000 Odense, Denmark

**Keywords:** cognitive behavioral therapy, blended treatment, depressive disorder, implementation, therapists’ perspective, routine care

## Abstract

(1) Background: Blended cognitive behavioral therapy (bCBT; online and face-to-face sessions) seems a promising alternative alongside regular face-to-face CBT depression treatment in specialized mental health care organizations. Therapists are key in the uptake of bCBT. This study focuses on therapists’ perspectives on usability, satisfaction, and factors that promote or hinder the use of bCBT in routine practice; (2) Methods: Three focus groups (*n* = 8, *n* = 7, *n* = 6) and semi-structured in-depth interviews (*n* = 15) were held throughout the Netherlands. Beforehand, the participating therapists (*n* = 36) completed online questionnaires on usability and satisfaction. Interviews were analyzed by thematic analysis; (3) Results: Therapists found the usability sufficient and were generally satisfied with providing bCBT. The thematic analysis showed three main themes on promoting and hindering factors: (1) therapists’ needs regarding bCBT uptake, (2) therapists’ role in motivating patients for bCBT, and (3) therapists’ experiences with bCBT; (4) Conclusions: Overall, therapists were positive; bCBT can be offered by all CBT-trained therapists and future higher uptake is expected. Especially the pre-set structure of bCBT was found beneficial for both therapists and patients. Nevertheless, therapists did not experience promised time-savings—rather, the opposite. Besides, there are still teething problems and therapeutic shortcomings that need improvement in order to motivate therapists to use bCBT.

## 1. Background

Face-to-face (FtF) cognitive behavioral therapy (CBT) is an evidence-based treatment for depression [1]. According to international guidelines, CBT is a standard option for treating patients with depression in specialized mental health care [2]. Unfortunately, patient access to CBT is hindered by several barriers such as perceived personal stigma, costs, long waiting lists, and limited availability of licensed therapists [3,4]. It seems therefore promising to offer CBT with the support of technology and without the FtF sessions, thereby potentially reducing therapists’ time and costs due to increased involvement of patients themselves. In Internet-based cognitive behavioral therapy (iCBT), treatment access can be improved to offer CBT on an online platform. This would help reduce stigma, waiting lists, and costs, since patients can work through sessions at their own pace, in their own time, and therapist time is lessened. It has been shown that iCBT, especially with online therapist guidance, has good clinical effects for patients with depression [5,6].

In geographically dispersed countries like Canada and Australia, online clinics have successfully reached many patients through iCBT (e.g., Mindspot Clinic, Online Therapy Unit) [7]. In the Netherlands, iCBT, with guidance at a distance, has been offered by Interapy for more than two decades (www.interapy.nl). This mental health care organization (MHO) was founded as an online therapy center and provides specialized online evidenced-based care for among other thing depression, trauma and panic disorder. Other MHO’s, where face-to-face treatments are the standard, also are implementing iCBT to improve patient access and make treatments more cost-efficient. However, iCBT uptake in MHOs is limited. This might be partly because therapists perceive iCBT to be limited in crisis situations, ineligible for severe depressive symptoms, and having a one-size fits all approach [8,9]. In addition, given the relatively high density of mental health services, the need for iCBT in the Netherlands may differ from elsewhere.

Blended CBT, a new format of iCBT, which has been recently adopted by MHOs in routine care systems in the Netherlands, could address the limitations since online sessions are combined with FtF sessions in a single, integrated, standardized CBT treatment protocol [10]. bCBT aims to improve both quality of care and cost-efficacy by replacing a portion of the FtF sessions with online sessions [11,12]. Through reducing the number of FtF sessions, therapists’ time is saved, they can potentially treat more patients, and waiting lists can be shortened. In addition, within the online platform, therapist contact is extended beyond the FtF sessions by online communication. A number of studies have shown that bCBT can be effective in treating patients with depression [13,14,15]. However, similarly to iCBT in MHOs, the uptake of bCBT by therapists and patients is limited as it has not yet been implemented across the board in MHOs in the Netherlands and elsewhere [16].

The gap between promising research findings and the limited iCBT and bCBT uptake in routine practice was one of the reasons behind the European MasterMind project, designed to increase and evaluate uptake and implementation of Internet-based interventions in ten European countries (www.mastermind-project.eu) [17]. In MasterMind there was a specific focus on therapists’ perspectives, as they play a substantial role in the uptake of iCBT and bCBT. And besides, studies concerning therapists’ perspectives in routine practice are scarce [18].

In a qualitative study in Germany, several barriers and facilitators were identified based on the perspectives of therapists (*n* = 5) providing bCBT alongside a randomized controlled study [19]. Key issues varied from problematic platform technology to an unclear concept of embedding bCBT in the mental health care system. Despite the barriers, therapists viewed bCBT as an adequate treatment option for patients with depression. To build on this, more in-depth knowledge on therapists’ perspectives in specialized routine care could contribute to optimizing uptake of bCBT, as the therapists’ workflow and patient-related factors are more complex in daily practice than in research settings.

The objective of this qualitative study was to investigate therapists’ perspectives in routine care when transitioning to using bCBT as an alternative alongside CBT for patients with depression within outpatient specialized mental health care settings in the Netherlands. To increase insight into the factors that influence uptake of bCBT in routine care, the following questions were answered using data triangulation [20] in the context of questionnaires, focus groups, and semi-structured interviews with therapists:

- What are the therapist perspectives on bCBT usability (e.g., perceived ease of use of the platform)?
- How satisfied are therapists with offering bCBT (e.g., overall satisfaction with bCBT)?
- What are the therapist perspectives on factors that promote or hinder the use of bCBT in daily practice (e.g., training, patient eligibility, safety)?

## 2. Methods

### 2.1. Therapists

Therapists were (CBT trained) psychologists, mental health nurses, or psychiatrists working at seven MHOs where bCBT had been implemented. To be included in the study, therapists had to be trained in the use of the online platform prior to or during the study period.

The therapists were employed at small (200 registrations for depression per year) to large private-non-profit MHOs (5000 depression registrations per year) spread across the Netherlands. All organizations had bCBT available on a platform from the same large commercial provider (Minddistrict; www.minddistrict.com)—most since 2014. MHOs provided bCBT training to (all or a part of) their therapists either ‘in-house’ or externally by the provider, or via online training. The estimated duration of the training varied from four hours to two days. MHO’s stimulated therapists in different ways for bCBT (e.g., by setting bCBT targets or by special blended consultation hours), however therapists could make their own decision about the treatment of choice.

### 2.2. Recruitment

Through other users of the Minddistrict platform and personal contacts of the authors, 10 MHOs were invited to be part of the study because they implemented an online platform into routine care. One MHO withdrew because it ceased to exist, another MHO was too busy with ongoing other projects and trials, and one MHO switched to a different platform provider and needed time to explore other providers. In total, seven MHOs participated.

Therapists were identified through team managers, and other specially members of different teams, appointed by the manager of the MHOs. Therapists for focus groups or in-depth interviews were purposively sampled to cover diversity of use and experience with bCBT, age, professional background, and geographic region. First, therapists from two MHOs were invited to participate in a focus group. The focus groups were held within the context of the European implementation project MasterMind. The MasterMind project ran from March 2014 to February 2017. For a more detailed description, see the study protocol of the MasterMind study in the Netherlands [18]. In total, 3 focus groups were held with 21 participants.

**Figure 1:**
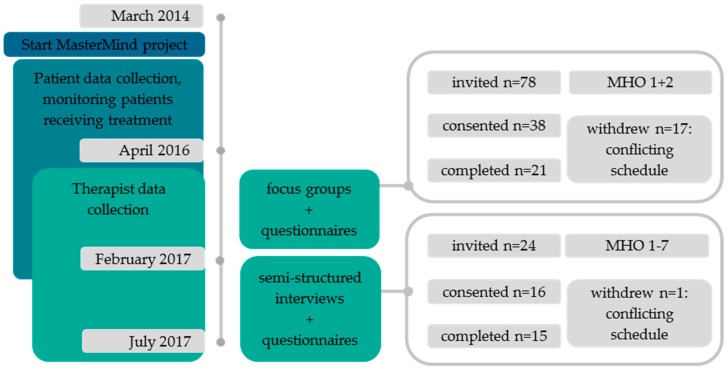
Study outline.

Second, to add more depth to the topics discussed in the focus groups, complementary semi-structured individual interviews were held with therapists (who had not participated in the focus groups) from seven MHOs. After 15 interviews, no new information or themes were brought up and data saturation was reached. Two weeks prior to the focus group and interviews, all participating therapists received an informed consent form and questionnaires were used to gather information on therapist demographics, satisfaction, and usability of bCBT via a secure online survey tool (Survalyzer; www.survalyzer.com). Figure 1 provides an outline of the study.

### 2.3. Intervention

Therapists worked with an integrated bCBT protocol in which FtF and online sessions were alternated. The blended protocol for depression mostly consisted of 10 online sessions. These were supported by FtF sessions (for mild or moderate depression 5 FtF sessions every two weeks; and for (very) severe depression 10 to 11 weekly FtF-sessions). bCBT started and ended with a FtF session. Therapists could individualize the protocol to the patient’s needs by repeating and/or skipping online sessions. More detailed information about the protocol can be found elsewhere [21].

Patients could be offered bCBT if (1) they were aged 18 years or older; (2) had a mild, moderate, or severe depression as a primary diagnosis according to the therapist; and (3) were indicated for cognitive behavioral treatment for depression following routine secondary mental health care procedures. Exclusion criteria were (1) not having a valid email address and a computer with Internet access and (2) not having adequate Dutch language skills (both verbal and written).

Therapists had access to the (Minddistrict) platform through a dashboard, which showed treatment sessions, including homework assignments, and had optional functions for a patient diary and depressive symptom questionnaires. The role of the therapists was to monitor the patients’ online progress and to provide patients with personalized written feedback to homework assignments after each completed online session. Therapists could additionally communicate through a message function on the platform about practical issues (e.g., upcoming appointments, reminders, or questions about assignments). The safety of the platform was guaranteed by meeting the European requirements for certified data security [22].

### 2.4. Data Collection

#### 2.4.1. Questionnaires

The 10 items of the System Usability Scale measured perceived usability (SUS) [23]. The SUS is a validated questionnaire for evaluating usability of Internet-based interventions as perceived by therapists and showed a good reliability (omega coefficient = 0.91) [24]. It is unofficially translated in Dutch [25]. The score for the total score range from 0 to 100. A score above the cut-off of 68 is considered to be above average [26]. The Client Satisfaction Questionnaire-3 measured satisfaction with bCBT (CSQ-3), is officially translated to Dutch [27] and was adapted to therapists (e.g., CSQ1 = To what extent has the bCBT intervention met your needs in treating depressed patients?). The CSQ showed good reliability (McDonald omega = 0.95) and validity in a sample of Internet-based depression intervention users [28].

#### 2.4.2. Focus Group Interviews

To identify relevant therapist factors in bCBT uptake, dimensions of the RE-AIM framework (Reach, Effectiveness, Adoption, Implementation, Maintenance) [29], the MAST framework (Model for assessment of telemedicine) [30], and the normalization process theory [31] were used to build and structure the focus group topic list (see File S1 in Supplementary Materials). The main predetermined themes were: patient and therapist factors, barriers and facilitators, satisfaction and usability. The focus group was pilot tested with a moderator and three participants who acted as therapists to refine the topic list and time management. Key questions were presented on a PowerPoint presentation during the therapist focus groups. Via a mobile voting system (Sendsteps; www.sendsteps.com), participants could give their opinion on certain questions and statements in order to stimulate discussion. Discussions were moderated by senior researcher and co-author S.D. with experience in conducting focus groups. They were assisted by researcher and first-author M.M. The focus groups were audio-recorded. The duration of the focus groups was 100–115 min and they took place at the participating MHOs. To increase validity, a transcript of each focus group was sent to each participant for feedback.

#### 2.4.3. Semi-Structured In-Depth Interviews

In semi-structured interviews, therapists were asked individually to reflect on the same topics as in the focus groups to further explore personal experiences. In addition, questions were added based on interview guides from previous research [19,32,33] as well as outcomes of the focus groups; there were slightly different questions for therapists with and without experience in providing bCBT for depression (see File S2 and S3 for the topic lists in Supplementary Materials). After 10 interviews, 3 additional topics were added since these seemed relevant to therapists (e.g., experience with writing online feedback, preferred ratio of online versus FtF sessions and preferred starting point for introduction of online platform). Interviews were conducted by two researchers (M.M. and C.v.G.). The interviews lasted 35–90 min. Most interviews were held at the MHOs (*n* = 10); five were conducted by telephone for practical reasons. The interviews were audio-recorded. An interview transcript was sent to each participant for respondent validation.

### 2.5. Data Analyses

Descriptive analyses (frequencies, means, and percentages) of the quantitative data (demographics of therapists, perceived satisfaction, and usability) were performed with SPSS, version 22.0 (IBM Corp. Armonk, NY, USA). Atlas-ti.7 software was used for qualitative analysis (version 7 and 8, Scientific Software Development GmbH. Berlin, Germany).

Focus groups and semi-structured in-depth interview transcripts were concurrently analyzed with thematic analysis techniques guided by the following steps [34]: (1) familiarization with the data; (re-)reading of the transcripts and field notes; (2) generating initial codes; developing a codebook; (3) searching for (sub)themes; identifying broad topics; refining codebook; (4) reviewing the (sub)themes; (5) defining and naming (sub)themes; and (6) reporting. Anonymity was assured by using code numbers instead of names. Each sentence in the transcripts could contain multiple codes, and concurrent sentences of the same thematic code could be conjoined into one unit.

In the results section, therapist’ quotes were selected to illustrate the essence of a (sub)theme. The statements reflected perspectives from both focus groups and interviews. We chose to pool these sources as similar topics were discussed and no overlap existed between the therapists who participated in the focus groups and interviews. Both contributed to a more comprehensive understanding of therapists’ perspectives. When a perspective concerned a minority of therapists, patients, or other therapists within the participating organizations, this is explicitly mentioned.

To ensure reliability, agreement on codes and concepts between members of the research team was sought. Researchers M.M., C.v.G., and S.D. coded 4 interviews together until acceptable agreement (Fleiss kappa >0.50) was reached [35]. The remaining interviews were divided equally between researchers M.M. and C.v.G. Data collection process, data analysis, and any interim findings, as well as quality and methodological aspects, were discussed in the research group continuously.

### 2.6. Ethical Issues

The study was approved by the Medical Ethics Committee of the VU medical center, Amsterdam. They confirmed that the Medical Research Involving Human Subjects Act does not apply (registration number 2014.580) because therapists in this study were not required to follow certain procedures on behalf of the research (no randomization), and routine practice was followed. An internal scientific research committee approved the research proposal.

## 3. Results

Table 1 presents therapist demographics and experience. With respect to distribution of experience with bCBT for depression, 42% of the therapists had considerable bCBT experience (providing bCBT 5 to 20 times or more), 42% had little experience (providing bCBT 1 to 4 times), and 17% had no experience.

### 3.1. bCBT Usability

The mean SUS total score was 69.5 (SD 11.1; range 42.5–90.0). This score indicates sufficient usability of the bCBT platform. In the focus groups and interviews, therapists indicated that the usability had evolved over time. However, the current technical status still needs further improvement. For some, platform usability was a barrier to providing bCBT because many extra actions were required (e.g., logging in, finding the module, setting up the sessions). In addition, there was no integration with other (administrative) computer systems that therapists frequently use (e.g., electronic medical records). Moreover, therapists felt hampered by the lack of knowhow and of a clear overview of all the functions and were unsure how to adapt the protocol. For example, it was unclear to them how to add sessions focused on panic attacks. Some therapists felt the platform usability was complicated and not intuitive. Unexpected updates by the provider did not improve this. For therapists with little bCBT experience, the concern that they would be unable to assist patients with technical problems was also a major barrier. In addition, therapists had negative experiences with contacting the helpdesk (e.g., lack of expertise): “I trained myself to not contact the helpdesk anymore” (T23, experienced).

### 3.2. bCBT Satisfaction

On the CSQ-3, 77% of therapists stated that bCBT met all or almost all their needs; 94% were overall very or mostly satisfied with bCBT; and 97% would recommend bCBT in the future to their patients. Interestingly, in the focus groups and interviews, the opinions of other therapists about satisfaction were divided: some thought that most therapists were generally satisfied, others said: “I also think that therapists are rather dissatisfied. bCBT isn’t used on a large scale. This means they must be dissatisfied, otherwise they would use it” (T28, no/little experience).

Most of the therapists indicated that they would like to use bCBT in the future, but this depended on several preconditions that needed to be addressed: platform usability, their current work routine, and more guidelines on how to use bCBT.

### 3.3. Factors That Promote or Hinder Uptake of bCBT

Regardless of the amount of bCBT experience, therapists were in favor of bCBT. Their views on the factors that promoted or hindered the use of bCBT in daily practice were structured along main themes identified in thematic analysis: (1) therapists’ needs regarding bCBT uptake, (2) therapists’ role in motivating patients for bCBT, and (3) therapists’ experiences with bCBT (See Table 2 for main and subthemes).

#### 3.3.1. Theme (1) Therapists’ Needs Regarding bCBT Uptake

This theme addresses factors related to therapists’ needs in providing bCBT to patients with depression. The identified subthemes were: therapist training, therapist motivation, and therapist readiness for uptake (Table 3).

##### Therapist Training

Most therapists found the training formats sufficient for providing bCBT. The focus in the training formats was primarily targeted at technical aspects and less at therapeutic content. The therapists reported that they needed more guidelines and tools for providing online feedback, to keep patients engaged and to prevent them from deviating from the protocol. The training formats in their current form were probably sufficient for the ‘early adopters’ but less suited for therapists with limited computer skills, the older generation, or new graduates.

Moreover, undergoing training alone was not enough. Therapists found it difficult to familiarize themselves with the platform, and integrate bCBT into existing systems and into their daily workflow. Time constraints were the most frequently cited barrier for uptake of bCBT. This was mainly due to a high caseload. Plus, therapists felt that the implementation ceased after the training was provided, and that the training was fragmented and was introduced too suddenly. Consequently, some organizations offered therapists a special consultation hour or extra training day. Yet, only a select group of motivated therapists turned up at these events. In order to integrate bCBT into their work routine, therapists needed more ongoing technical and content support in the form of supervision or peer review.

Above all, every qualified therapist was considered able to provide blended treatment, mostly because bCBT was perceived as being very similar to regular CBT. However, they also believed that therapist qualifications and professional background have so far been overlooked. They felt sufficient knowledge and professional experience of CBT should be a criterion for being trained in bCBT.

##### Therapist Motivation

Therapists in this study were motivated for bCBT. Their colleagues could roughly be divided into three groups based on their motivation: demotivated therapists, motivated therapists, and an inexperienced group that needed more motivation.

Therapists estimated that about half of their therapist colleagues were not motivated for bCBT. They thought that perceived pressure from the MHOs and health care insurance companies worked counterproductively. Other reasons were varied. Some thought that a number of colleagues tended to resist every innovation implemented by the organization. For others, the reasons were fear-based: (1) fear that technology will eventually take over, (2) fear of losing contact when a patient is inactive on the platform, (3) fear of not offering patients the amount of help they need, and/or (4) fear that they would do something wrong or would come across as unprofessional towards patients. A specific group of therapists, who were enthusiastic at the start but due to negative experiences with (other/outdated/unguided) Internet-based interventions, lost interest or became sceptical about the clinical effects of bCBT. This was mainly due to technical issues and higher patient drop-out rates.

Motivated therapists were described as being younger, having affinity with technology, and being interested in innovations in their field. Not every therapist was open to the idea of bCBT in the beginning, but some adjusted their view based on (positive) experiences.

Non-experienced therapists were mainly profiled as lacking computer skills, having technical skills insecurities, and belonging to an older generation with a traditional view on therapy. Interestingly, it was stated multiple times that many therapists were open to bCBT and also were capable of delivering it, but had reservations that first needed to be addressed. On top of a strong preference for FtF treatment over bCBT, there are thought to be many therapists to whom the possibilities of bCBT were unknown or remained unclear. They were often unaware of its added value or of research findings. For them, the need for blended did not come from within. However, even though there might be many reasons not to provide bCBT, therapists believed that only a small proportion are truly resistant to it.

##### Therapist Readiness for Uptake

Though most therapists received training on the back of an organization-wide roll-out, a number observed that only a select group used bCBT on a daily base. To some, this was related to readiness for uptake: the implementation of bCBT in routine care is considered to be at an early, transitional stage. Although it might be too innovative for therapists and patients, they believed that a change in perception toward bCBT will take place, and that readiness would increase as more patients start asking for bCBT.

There was disagreement with respect to making bCBT obligatory or not. For some, it would feed resistance toward bCBT and fuel the suspicion that bCBT was solely implemented for financial reasons. Others believed that every therapist should ‘just use it’ or at least try it. It was also felt that treatment should be ‘blended, unless a therapist has limited computer skills, or a different therapeutic background or interest’. Most therapists stressed that for them, the precondition that blended stays blended is of great importance, because of the fear that MHOs in the future might gradually transition to online CBT.

#### 3.3.2. Theme (2) Therapists’ Role in Motivating Patients for bCBT

The second theme concerns the role of therapists in motivating patients with depression to use bCBT. The identified subthemes were: informing patients, patient eligibility, and patient resistance (Table 4).

##### Informing Patients

Many therapists had difficulties informing patients about bCBT for various reasons. Reasons given included the following: they were convinced that patients preferred FtF contact; blended was considered too demanding for patients; or they thought that patients would not need bCBT. Therapists thought that more effort could be put into convincing patients since the manner in which bCBT is offered could influence patients’ reactions.

Therapists found it a disadvantage that patients did not ask for bCBT or lacked a clear idea of what blended entails. Therefore, therapists believed it could be facilitating to offer it with enthusiasm, to explain the added value: ‘patients can do more in their own time, there is more information to their disposal, and they have more opportunities to practice, all in a secure environment’. It was indicated that there is a lot of room for improvement when it comes to informing patients. It helped, for example, if patients knew that it is their own therapist who communicates with them on the platform. In addition, to increase uptake, therapists should not offer it as a treatment option, but as the standard approach to treating depression. Some therapists offered blended as something extra, whereas others showed patients the platform during intake to facilitate the switch to the platform at a later point during treatment.

##### Patient Eligibility

Experienced therapists mentioned that the criteria for bCBT were very similar to the criteria for CBT. No specific patient bCBT profile or type existed; eligibility was found to be person specific, not predictable and sometimes even very surprising. Therapists with little or no bCBT experience gave many different criteria for patient eligibility, which were predominantly based on perceptions.

A contributing factor to difficulties with informing patients was the unknown typology of the eligible bCBT patient. Stated criteria for perceived non-eligibility were extensive: lack of computer skills, preference for FtF treatment, fear of failure, lack of illness insight, low cognitive capacities, limited language skills, insecure home environment, psychotic symptoms, suicidal ideation, personality disorder, trauma, and complex or severe depressive symptoms. Perceived eligible patient criteria were less elaborate: young age, having children, being employed, and mild to moderate depressive symptoms.

Two interesting discussions took place about patient eligibility. Therapists disagreed about patients who, besides depression, had autism, avoidance issues, limited social contacts, poor concentration skills, or had undergone CBT for depression in the past. Some therapists observed that these factors prevented patients from engaging with bCBT, whereas others experienced that these factors made bCBT explicitly eligible for these patients as well. Another point of discussion was the severity of the depressive symptoms. Some therapists thought that bCBT was suitable for patients with complex, severe problems, while others did not: it would be harder to activate them than patients with less complex and more moderate symptoms. They would need more tools and explanations than bCBT can possibly offer them.

##### Patient Resistance

Several reasons for patient resistance toward bCBT were observed: unclear bCBT image, misfit with the traditional image of therapy among patients who had CBT in the past, fear of not receiving the right amount of help, and thinking that the online component was too demanding. Therapists’ found that some patients were more difficult to motivate because they were inclined to generalize negative experiences with previous Internet-based treatments (often unguided treatments in primary care) to all other Internet-based interventions. However, most therapists, especially the experienced ones, thought the vast majority of patients were positive about bCBT when offered. Most therapists agreed that patient demand will increase in the future.

#### 3.3.3. Theme (3) Therapists’ Experiences with bCBT

Within this theme, experiences with providing bCBT in routine care are discussed. The identified subthemes were: effectiveness for depression, positive effects, negative effects, treatment format, therapeutic relationship, online feedback skills, drop-out, and safety (see Table 5).

##### Effectiveness for Depression

Therapists agreed that bCBT offered a good foundation for treating patients with depression. Nevertheless, they also found that the therapeutic content of the online sessions needed improvement, especially for patients with severe depressive symptoms who were characterized by more inactivity, complex problems, and longer treatment history. Whether ‘the blended’ treatment format exists, remained a question. Not based on research findings, but based on their experience, therapists uniformly thought that bCBT and FtF CBT could be equally effective. bCBT mainly helped to structure treatment and both patient and therapist to remain focused.

##### Positive Effects

The most cited positive effect was containing therapist drift; platform functionalities facilitated therapist (and patient) focus and protocol adherence more than in regular CBT because of the pre-set structure of bCBT. Therapists found it easier to control homework assignments and patients knew what was scheduled next. It was believed that all this positively impacted the quality of the treatment. An additional effect was that other diagnoses besides depression (e.g., autism) or certain problems (e.g., suicidal ideation, non-adherence, low cognitive capacities) were made more visible to the therapists at an earlier treatment stage. This was due to a discrepancy between online symptom monitoring, patients’ online expressions, and what was said and shown in FtF sessions. Plus, for therapists, it was more noticeable from the patients’ writing skills what the cognitive abilities were.

In the therapists’ experience, patients found it easier to remember what was discussed in FtF sessions with bCBT. The information on the platform supported therapists—they had more information available, not only about depression but on other treatment options as well (e.g., sleep, self-image). In addition, because patients with depression often have concentration problems, the unlimited and easy access to treatment information and psychoeducation was very useful.

Reducing travel-time was mentioned as being very convenient for patients, not only for practical reasons (e.g., having children, employment), but also for therapists. bCBT provided an extra tool to stay connected with patients, especially in cases of no-show. Therapists discovered that patients shared the platform content with their immediate circle more easily (e.g., partner, family, or friends). Plus, the effect of writing on the online platform was a big advantage to patients. Patients wrote down their problems, came to new insights, and shared shameful problems more easily because of the distance effect and having time for reflection. bCBT offered patients greater responsibility. As for self-efficacy, some therapists thought that patients tended to attribute the treatment success to themselves more than in FtF treatment.

##### Negative Effects

Most therapists questioned whether bCBT could shorten treatments and solve waiting list problems. First, therapists spent a lot of time getting to know the platform and secondly, providing therapy via the online environment was time-consuming in contrast to expectations. Perhaps it might be timesaving in the future. However, the common thought was that bCBT is ‘just another way of doing the same thing’. Some felt that there is a long way to go before blended interventions are considered equally effective as regular CBT by therapists in general.

Some judged the content on the platform to be too textual, too complex, or too superficial, thus requiring repeat sessions and extra clarification. Moreover, sometimes patients did not recognize themselves in the text and video examples; some found the content pejorative, overly directive, or too intense, resulting in drop-out or even worsening of their mood. Therefore, some therapists felt a strong need for personalization of the content to adapt to the patients’ preferences and background.

##### Treatment Format

While experienced therapists unanimously stated that the pre-set structure of the blended protocol was very beneficial, for therapists with less experience, this was not the case. For them, the protocol was too structured and therefore inflexible. Experienced therapists, who had a better overview of all the functionalities, mentioned that bCBT was very similar to FtF CBT, in which you also adapt to the protocol when needed.

Therapists had different views on the ratio of FtF versus online sessions within bCBT. Some regarded and used bCBT as an addition to the regular number of FtF sessions. Moreover, for some the question ‘what is blended?’ remained. Therapists had different views on degrees of ‘blendedness’. Repeatedly, it was seen as an interchange of FtF sessions with the functionalities of the online platform. For some patients 50%–50% was considered beneficial, for others 90% FtF and 10% online was more appropriate.

Another issue was the time point at which the online platform was introduced. Some therapists preferred to start with weekly FtF sessions and introduced the platform at a later date. For them, it felt unnatural and too distracting for patients to meet every two weeks instead of weekly in the first phase of treatment. They wanted more time to establish a foundation, especially for patients who lacked insight into their depression. Other therapists, who thought it would be better to start with the online platform at once, reasoned that the threshold (for patients and therapists) may be higher when the platform is introduced at a later stage. These therapists also imagined that it motivated patients to be focused from the beginning with the support of the platform.

##### Therapeutic Relationship

Regarding the quality of a therapeutic relationship in bCBT, therapists’ opinions were divided. In the beginning, some doubted whether it was possible to develop a therapeutic relationship because they would see patients less FtF, but changed their opinion later on. In some therapists’ experience, it was challenging to build a therapeutic relationship compared to regular CBT; when a patient experienced difficulties with the online platform and asked more attention from the therapist, this frustrated the contact because it was at the expense of the time they had FtF. Nevertheless, others experienced that the relationship in bCBT was similar or even better compared to FtF treatment. bCBT reinforced the relationship, since there was more frequent contact throughout. In addition, the treatment course was more visible to therapists because of online monitoring and this also positively influenced the relationship. Importantly, as in every treatment, the relationship was mainly perceived as more dependent on patient motivation and activation than other factors such as online contact.

##### Online Feedback Skills

Therapists agreed that every therapist is able to provide online feedback. However, learning how to provide feedback took time and for many, felt like ‘a job in its own right’. They were needed for tips, tools, and examples; how to address something negative, how to handle or prevent miscommunication, how to write in a concise manner, how to make feedback personalized and adapt to different patient types. In contrast to FtF sessions, where therapists are able to correct themselves, everything that is written online, has a permanent character: for some, online feedback thus felt uncomfortable.

Therapists stressed the importance of connecting to the language of the patient. They found they had to be careful not to use difficult terminology or be too distant in their language. Most of all, they had to plan sufficient time to write feedback. Therapists noticed that writing online feedback became faster with more experience. Connecting online feedback and topics in FtF sessions and vice versa, was experienced as helpful and was done quite frequently.

##### Drop-Out and Safety

Two safety risks cited predominantly by inexperienced therapists were the suggestion of 24/7 accessibility to patients and not being able to see how patients reacted to therapist feedback or other online information. Experienced therapists did not mention this or had learned that this was not an issue.

Regarding a much-discussed risk of suicidal ideation, therapists experienced that when there was a clear agreement with patients on not expressing crisis situations on the platform, bCBT had no extra risks compared to FtF treatment. Importantly, it even gave therapists more rapid awareness of whether patients were losing contact. In addition, bCBT provided therapists with an extra preventive communication tool.

Furthermore, experienced therapists reported that there was no specific type of patient that dropped out. Sometimes therapists saw patients who started enthusiastically, but dropped out easily. Importantly, they thought this was no different than with regular CBT: patients dropped out because of certain patient characteristics or other problems than for bCBT-related issues.

Overall, therapists expressed that safety of patient data on the online platform is guaranteed sufficiently. Sharing patient data with, for example, a helpdesk, is still something that therapists were reluctant to do and prevented them from contacting the helpdesk when technical issues occurred. They often then tried to solve their issues with colleagues, but often also broke off the online sessions. Some patients seemed too lax when it came to their data safety, and some were distrustful. Therapists emphasized that it is their responsibility to explain how safe the online platform is, but that this was not clear to all therapists.

## 4. Discussion

In this study, therapists expressed overall satisfaction with providing bCBT to patients with depression. The perceived usability of the online platform was sufficient. This was also found by other studies exploring patients’ perspectives on usability and satisfaction [21,36]. This study showed that therapists see room for improvement with regard to platform usability (e.g., lack of integration with other administrative systems), therapists’ work routines, and guideline use on bCBT. From interviews in this study, it became clear that there are specific barriers that prevented therapists from providing bCBT to patients with depression, such as a lack of ongoing support for technical and clinical issues with bCBT. In addition, therapists reported several practical and therapeutic challenges in routine care such as not experiencing timesaving. Nevertheless, there have been factors that might have influenced the uptake positively as well.

### 4.1. Barriers to bCBT Uptake

Less experienced bCBT therapists felt several barriers prevented them from providing bCBT. First, they found that the training was not sufficient to enable them to adopt bCBT into their work routine. This was partly because the training mainly covered technical aspects and did not provide sufficient guidance on how to work in a blended way with the protocol and how to communicate online. In addition, after undergoing training, ongoing support and attention for bCBT was limited or absent. This was also a reported therapist barrier by Folker et al. [37] who identified implementation challenges perceived by therapists and managers of iCBT in routine care settings in five European countries. Moreover, inexperienced therapists were unsure about the indication for bCBT and the eligibility of the patients. A third issue was that some therapists experienced low patient demand for bCBT. A fourth hindering factor was that for a number of the therapists, the added value of bCBT in terms of clinical effectiveness remained unclear; for others, fear (e.g., of doing something wrong) or distrust (e.g., regarding the intentions of health insurance companies) stood in the way of considering bCBT.

### 4.2. Challenges with bCBT

Once therapists started working with bCBT, they experienced several challenges. They had difficulty integrating bCBT into their daily workflow. Not only because of technical disconnections with existing IT-systems for patients’ administration, but also because of uncertainties regarding the bCBT protocol and logistic integration into their daily therapeutic and administrative schedules. Besides, therapists lacked the conviction that bCBT saved time; for some, it even generated a higher workload, especially in the beginning, and providing online feedback is considered time-consuming as well. It can be estimated, that on average per feedback message, 30 min of therapist time is needed [13]. Internal helpdesks were unable to support therapists sufficiently which even discouraged some from further providing bCBT.

Moreover, therapists displayed different interpretations of the protocol which partly resulted for some in using the online platform on top of the FtF treatment instead of replacing a portion of the FtF sessions. This was previously reported in a naturalistic study on bCBT uptake in routine care as well [38], making the implementation more costly than intended. Kenter et al. [38] argued that their therapists may have needed more extensive training to master specific bCBT skills (e.g., providing online feedback) and that clear guidelines on how to use bCBT in routine practice by the MHO would have helped the therapist to provide bCBT more efficiently. Plus, using the online platform on top of the FtF treatment may result in patient dissatisfaction. A study on patients’ experiences with bCBT showed that a diminished interplay between the online and FtF sessions was unsatisfactory for patients, as therapists were less aware of patient activity on the online platform and limited time was made available to discuss the online activity in the FtF sessions [39].

### 4.3. Advantages of bCBT

One of the main reported advantages of bCBT was the focus and pre-set structure that made therapists and their patients more adherent to the protocol and contained therapist drift. This was also experienced by several therapists who worked with bCBT [14,19] or blended group therapy [40] for depression. Although content improvement (e.g., online patient examples) for severely depressed patients was considered necessary, therapists agreed that bCBT is a good treatment format for the (complex) patient group therapists see in daily practice. They found that bCBT was in many ways similar to FtF CBT when it comes to patient eligibility, effectiveness, therapeutic relationship, and drop-out: ‘it is CBT in another format’. Many believed that the implementation phase is still in its infancy; it might be a long way to go before all therapists accept blended interventions as being equally effective to FtF therapy. This idea is also partly justified since the first studies on clinical effects on blended interventions show that it is at least as effective [12,13]. Nonetheless, it is thought that bCBT will eventually be included in care pathways in the future because of the perceived benefits for patients and therapists (e.g., pre-set structure, number of flexible contact moments on demand, information access); an important precondition is to continue to be combined with FtF contact, as providing stand-alone guided iCBT might not yet be realistic for most therapists in routine practice, where FtF treatment is the norm.

### 4.4. Level of Experience

In our study, therapists with bCBT experience reported different views on safety, flexibility and personalization of the protocol, patient eligibility, and therapeutic relationship than therapists who lacked experience. For example, non-experienced therapists assumed that there were many reasons why patients would be ineligible for bCBT, such as severe depressive symptoms, or found this difficult to assess. By contrast, more experienced therapists stated that it was impossible to predict eligibility; in principle, any patient could be offered bCBT. This may indicate that with experience, perceptions on these important clinical factors of bCBT will change and that when promoting uptake, therapists’ needs can shift. This is consistent with Feijt et al. [41] who showed that there are different barriers and facilitators depending on the level of therapist experience with online services, and that potentially, the experience level has unique requirements to be addressed when it comes to the uptake of Internet-based interventions.

### 4.5. Limitations and Strengths

Although some therapists in this study were skeptical about the effectiveness of bCBT or had negative experiences, all were quite open-minded toward bCBT. Selection bias in our sample is possible, as untrained therapists or those not interested in participating in the study were not reached and therapists were identified through their team managers, and other specially appointed members of different teams. This could have diminished the positive view on bCBT.

Another point is the limited transferability of our findings to other national contexts. In the Netherlands, the digital infrastructure is quite good and MHO’s and therapists have the experience that every patient has proper equipment. Moreover, bCBT is covered by health care insurance companies in the same way as face-to-face CBT. Compared to other countries, the Netherlands and others, including Sweden and the United Kingdom, can be considered a frontrunner in implementing bCBT within routine practice [16]. On the other hand, our findings can be taken into account by the ‘followers’ in the earlier stages of developing, testing, and implementing bCBT.

A strength of this study is the usage of data triangulation to record the perspective of a diverse group of therapists, with different professional backgrounds, differences in experiences with bCBT for depression, and exposure to various implementation strategies from a number of mental health care organizations.

### 4.6. Future Uptake of bCBT

To speed up bCBT uptake, it is important to identify how barriers can be overcome, challenges solved, and how successes in routine care can be strengthened. From the field of implementation research, it is known that implementation strategies for innovations in mental health care need ‘to address multiple levels and barriers to change, (and) are interwoven and packaged as a protocolized or branded implementation intervention’ [42]. Based on this and on the findings of this study, the following strategies can be considered in practice and in research to move forward in the implementation of bCBT:

1. Dissemination strategies: to change the focus of cost-effectiveness of bCBT towards personalization, adherence, and treatment quality improvement in disseminating information to therapists and patients. Within organizations, the uptake might gain from informing and motivating therapists and subsequently patients as well. There is a lot of room for improvement when it comes to sufficient knowledge about bCBT. Outside the organizations, patient demand can be stimulated by general practitioners and health insurance companies. Further, although evidence-based knowledge on bCBT is slowly becoming more available, this should be more widespread among patients, therapists, MHOs, and health insurance companies.
2. Top down strategies: bCBT must be embedded top-down from start to finish overcoming its mere project status in many of the MHOs. This could be achieved by intensifying and extending training beyond technical aspects, and by facilitating the integration of ongoing support into existing consultation structures, (instead of creating special consultation options), in order to exchange knowledge and experience with bCBT. It would also help to remind therapists to offer bCBT to patients and to solve technical disconnections with administration- and other IT-systems within a well-equipped help desk. Whether imposing minimum usage norms (e.g., that 25% of the treatments must be blended in the Netherlands) could have a promoting effect is unclear, as this causes resistance for some, but motivated others.
3. Bottom-up strategies: Fragmented use and individual uptake of the blended protocol can create a risk of it being costlier and of leading to a protocolled manner of working. Therapists’ need to personalize modules might be a result of uncertainty and their limited knowledge of all functionalities within the module and platform. Thus, besides a top-down approach, therapists might be more motivated and skilled if uptake is stimulated on a bottom-up basis, through discussing it on the work floor: For instance, it could help to start implementation with a small rollout with a motivated team of therapists, focusing on content development, enhancing online feedback skills and protocol use; to let the therapists be (co-) creators of modules; to construct a place and time within MHOs where therapists can think of ways to further develop bCBT; to experiment; to keep lines with the technical innovations on the platform within reach; including recurring evaluation in order to adapt strategies on the level of therapist experience and updates of the platform.

### 4.7. Future Research

Testing strategies for uptake would be a fruitful area for further research. The European project, ImpleMentAll (www.implementall.eu), will hopefully provide routine practice with more knowledge as this is uncharted territory for Internet interventions in mental health care [43]. In ImpleMentAll, personalized implementation strategies will be tested to facilitate the use of Internet-based and blended interventions in routine care in several different countries.

In addition, it would support uptake of bCBT to further investigate the ratio and integration of online and FtF sessions. So far, research has focused on an equal distribution. However, it is reasonable to think that depending on the severity of the symptoms, the proportion of blended could be adapted. Moreover, it would be interesting for future studies to also look at the patient perspectives on blended care, preferably done using dyad interviews with their therapists to generate a rich, deeper understanding of the relevant factors in bCBT uptake.

## 5. Conclusions

Overall, the therapists in this study were satisfied with providing bCBT to patients with depression. That said, a large group of therapists is still wondering how much FtF contact should be included in blended. Plus, important preconditions for implementation were unmet and the technical infrastructure is not free from teething problems. It cannot be expected from therapists that uptake happens spontaneously. It can be considered positive that having bCBT experience can be a possible key to change a therapist’s view on important factors such as eligibility and drop-out. Therapists found that there is a good base that can be further developed in terms of ongoing technical support, therapeutic support from their peers and supervisors, while at the same time strategically implementing bCBT on an organizational level to facilitate therapists in the transition of integrating bCBT into their daily practice.

## Figures and Tables

**Table 1:** Therapist demographics and experience.

Therapist Demographics	*n* = 36
Gender, female, *n* (%)	29 (81)
Age in years, mean (SD; range)	38 (9.5; 24–60)
Professional background, *n* (%)	
Licensed psychologists	20 (56)
Psychologists in training under supervision for health care psychologists	5 (14)
Mental health nurses	5 (14)
Other (e.g., psychiatrists, prevention workers)	6 (16)
Professional experience, *n* (%)	
0–2 years	3 (8)
3–4 years	8 (22)
5–9 years	10 (27)
10 years or more	15 (42)
bCBT depression treatment experience, *n* (%)	
0 times	6 (17)
1–4 times	15 (42)
5–9 times	8 (22)
10–19 times	2 (6)
20 or more times	5 (14)

**Table 2:** Therapists’ blended cognitive behavioral therapy (bCBT) uptake in routine practice, main themes, and subthemes.

Theme (1) Therapists’ Needs Regarding bCBT Uptake	Theme (2) Therapists’ Role in Motivating Patients for bCBT	Theme (3) Therapists’ Experiences with bCBT
Therapist training Therapist motivation Therapist readiness for uptake	Informing patients Patient eligibility Patient resistance	Effectiveness for depression Positive effects Negative effects Treatment protocol Therapeutic relationship Online feedback skills Drop-out and safety

**Table 3:** Theme (1) Therapists’ needs regarding bCBT uptake, subthemes, influencing factors, and illustrative quotes.

Subtheme	Influencing Factors	Illustrative Quotes
Therapist training	(+) sufficient to learn the technical aspects	*“It is nice to have learned beforehand where all the buttons are and what the possibilities are.”* (T11, no/little experience)
	(−) lack of therapeutic content and guidelines in the training	*“I miss therapeutic content in the training and the qualifications for the therapists to work with it.”* (T25, experience)
	(−) training alone is not enough for uptake in daily practice	*“There is one training and then you’re released with the notion: ‘now you can manage everything’.”* (T7, experience)
	(−) lack of ongoing support	*“There is a lack of time to delve into it, it should be facilitated better and not end with a single training.”* (T14, no/little experience)
Therapist motivation	(−) demotivated therapists	*“What bothers me most when it comes to blended is that it has been made mandatory, mainly because we’ve to meet a certain percentage of blended treatments.”* (T34, experience)
	(+) motivated therapists	*“If you are a psychologist, and you are interested in your profession, you should just use it.”* (T9, experience)
	(−/+) undecided therapists	*“You have to get out of your comfort zone to discover what the added value of blended is.”* (T31, no/little experience)
Therapist readiness for uptake	(−) small select group uses bCBT on a regular basis	*“When I look at my team, it seems only one person is trained, I don’t really hear others talk about it.”* (T36, experience)
	(+) expected to grow in future	*“I’d like to use blended in the future, but first I’d like more ideas on how to use it* (T6, no/little experience)
	(−/+) making bCBT mandatory	*“I once heard that you need a certain percentage of online contacts—that would work for me.”* (T2, experience)

Note. (−) factor that influenced the bCBT uptake negatively, (+) factor that influenced the bCBT uptake positively, (−/+) factor that influenced the bCBT uptake positively and negatively.

**Table 4:** Theme (2) Therapists’ role in motivating patients for bCBT, subthemes, influencing factors, and illustrative quotes.

Subtheme	Influencing Factors	Illustrative Quotes
Informing patients	(−) difficult to motivate some patients	*“Sometimes there is so much going on, which leads me to inform patients in an insecure or doubtful way, that makes them say ‘no, I don’t want that’.”* (T35, no/little experience)
	(−) room for improvement	*“I think that therapists should ‘sell’ or promote bCBT more with patients.”* (T15, experience)
Patient eligibility	(−) eligible patient unknown	*“It is difficult to assess for whom it is eligible. Sometimes someone who grew up with computers says, ‘oh no, not for me’, and then a 65-year old says ‘nice, let’s do it’.”* (T10, experience)
	(−/+) discussion on comorbidity/complexity/depression severity	*“I imagine that blended is less suitable for more complex cases. But if I consider what I have in my caseload now, I think it should be doable.”* (T5, no/little experience)
	(+) eligibility bCBT = eligibility CBT	*“In principle everyone is eligible for bCBT. I rarely think that it’s not an option.”* (T7, experience)
Patient resistance	(−) unclear image, too demanding, not enough, negative past experiences	*“Patients don’t have a good image of bCBT. In their view it’s more like an online questionnaire than a complete module, ‘I’ve already answered those questions…’.”* (T9, experience)
	(+) patient demand expected to increase in the future	*“I think it will make an essential difference if patients ask for it. That’s just a matter of time’.”* (T1, no/little experience)

Note. (−) factor that influenced the bCBT uptake negatively, (+) factor that influenced the bCBT uptake positively, (−/+) factor that influenced the bCBT uptake positively and negatively.

**Table 5:** Theme (3) Therapists’ experiences with bCBT, subthemes, influencing factors, and illustrative quotes.

Subtheme	Influencing Factors	Illustrative Quotes
Effectiveness for depression	(+) effectiveness bCBT = effectiveness of CBT	*“It is CBT in another format, it’s not suddenly a different treatment.”* (T29, experience)
Positive effects	(+) containing therapist drift	*“I don’t skip things. With bCBT, I think that I am much more thorough than without.”* (T29, experience)
	(+) making other diagnosis or problems more easily visible	*“You notice much sooner if a patient has difficulties adhering to therapy, whether or not they are doing the work. This becomes clearer faster than in a FtF session.”* (T1, no/little experience)
	(+) helps to remember information more easily	*“In a FtF session you say a lot, but afterwards patients also forget quickly. Now they can review what they’ve learned as many times as they want.”* (T10, experience)
	(+) reducing travel time	*“In other countries, factors such as distances and climate play a role, but of course in the middle of the country we have traffic jams. bCBT is very nice for this.”* (T9, experience)
	(+) more contact with patient	*“Throughout the treatment you have more frequent short contact moments, which are actually very good.”* (T10, experience)
	(+) sharing content with system	*“For the patient you remove the treatment from therapist’s office to their own home.”* (T12, no/little experience).
	(+) effect of writing	*“Patients have to write, this is for many a very good instrument to order things and gain new insights. I find the writing a very positive addition to the verbal component.”* (T15, experience)
	(+) monitoring homework	*“I find it very attractive that there is more control over the homework that they have or haven’t done.”* (T2, experience)
	(+) facilitating patient self-efficacy	*“In the online part a degree of independence makes patients themselves engage with the treatment. I think that they attribute treatment success to themselves, more than with FtF treatment.”* (T22, experience)
Negative effects	(−) no gain in time, costs more	*“I find it takes more time than I expected. Especially if you’re still unfamiliar with the module.”* (T7, no/little experience)
	(−) unsuitable content	*“I had a traumatized patient, because she couldn’t have children. The first example on the platform was ‘I have children and I want to be a good mother...’.”* (T27, no/little experience)
Treatment format	(−/+) discussion on structure protocol	*“I find it difficult to be flexible in the protocol and not to become a sort of CBT machine.”* (T21, no/little experience)
	(−/+) discussion on freedom in protocol to explore depression	*“Usually, when I start without the platform, I think ‘never mind’. But also, when the patient isn’t ready for it initially, you can say ‘Later on, we will start with the online platform’.”* (T31, no/little experience)
	(−/+) discussion on ‘what is blended’	*“I don’t think it’s 50/50. The main focus is more on the FtF sessions. I notice that the conversations and the modules do not always run parallel to each other.”* (T14, no/little experience)
	(−/+) discussion on introduction platform	*“Whether you start treatment with an online module, right at the outset or only when a basis has been formed, is dependent on the therapist. I think am very comfortable with starting right away.”* (T1, no/little experience)
Therapeutic relationship	(−/+) discussion on quality therapeutic relationship	*“It’s possible to build a therapeutic relationship, but sometimes it isn’t. Yet I think that this isn’t dependent on bCBT. Also, with FtF therapy it’s possible to succeed in building a therapeutic relationship and sometimes not.”* (T15, experience)
Online feedback skills	(−) time providing feedback (−) permanent character of online feedback	*“I reflect more on what I want to write, that is perhaps the reason why it takes more time.”* (T7, no/little experience) *“It’s difficult to assess whether someone has understood something in the way you intended. If this happens in FtF contact you can talk around it, look friendly and explain. In black and white this is not possible.”* (T5, no/little experience)
	(+) connection online feedback and FtF conversations	*“I notice that patients like to come back to a point during the conversations, to go through the exercises together.”* (T12, no/little experience)
Dropout & safety	(−/+) discussion on safety risks	*“In the meantime, I experienced that it’s okay if the patient also comes to the FtF sessions. You’ve to make this clear at the start. The risk you lose contact is lower than I expected.”* (T9, experience)
	(+) bCBT has no extra risks with regards to suicidal ideation	*“I thought that if you see someone less that it’d be more difficult to assess the suicide risk. But it can just as well lower the threshold to say something online more easily than to discuss FtF.”* (T31, no/little experience)
	(+) drop-out bCBT = drop-out CBT	*“I don’t have one type of patient that drops out of blended. It’s very unpredictable who completes it.”* (T30, experience)
	(+) sufficient patient data safety	*“I understood that the online platform is very safe, safer than email.”* (T33, experience)

Note. (−) factor that influenced the bCBT uptake negatively, (+) factor that influenced the bCBT uptake positively, (−/+) factor that influenced the bCBT uptake positively and negatively.

## Supplementary Materials

The following are available online at https://www.mdpi.com/2077-0383/9/1/91/s1, File S1: Topic list focus groups, File S2: Topic list semi-structured interviews experienced therapists, File S3: Topic list semi-structured interviews non-experienced therapists.
