# Supplementary material for: Why Uptake of Blended Internet-Based Interventions for Depression Is Challenging: A Qualitative Study on Therapists’ Perspectives
Source: J Clin Med. 2019 Dec 30;9(1):91. doi: 10.3390/jcm9010091 (PMC7019532; doi:10.3390/jcm9010091)
Supplement: Supplementary file 1 [file jcm-09-00091-s001.pdf]

File 1: Topic list focus groups

1. Introduction

This focus group is being held in the context of the MasterMind research. MasterMind stands for: MAnagement of mental health diSorders Through advancEd technology and seRvices-telehealth for the MIND. MasterMind is a large European study. It is an implementation study in which cohorts of patients are followed in 11 countries who receive Internet treatment for depression. Expectations are high and the results are promising, but Internet treatments are still used relatively little in practice. We therefore want to investigate how this treatment can best be applied, which factors are facilitating, and which ones actually get in the way of implementation.

In the Netherlands, we are investigating how blended CBT treatment is applied in basic and specialist mental healthcare for patients with depression older than 18 years. Data is collected from 300 patients, practitioners, and the representatives of the mental healthcare organizations, including your organization.

The aim of the focus group is to provide insight into the experiences and ideas of you as a blended therapist.

1.1. One word

- [Question mobile voting system] What do you think is, in one word, the most important thing when implementing eHealth in mental health care (open answer)?

1.2. Patient factors

- [Statement mobile voting system]: Patients need the blended treatment for depression (yes/no).

The patient's need:

- Which needs of the patient does the blended treatment meet?
  - What is the most important aspect of this?
- What makes the blended treatment attractive to patients?
- Which needs of the patients does blended treatment not meet?
- [Question mobile voting system]: How suitable is the blended treatment for patients with depression? (number 1-10)

Patient profile:

- Which patients start this treatment?
- Which patients can benefit from the blended treatment?
- Which patients drop out with the blended treatment?
- What are the most common reasons for patients not to complete the blended treatment?
- [Statement mobile voting system]: There are many safety risks for the patient associated with the blended treatment (yes/no).

Safety

- What are the risks associated with blended treatment?
- Is the safety of the patient sufficiently guaranteed?

1.3. Therapist factors

- [Statement mobile voting system]: Therapists need the blended treatment for depression (yes/no).

The therapist's need:

- Which needs of the therapist does the blended treatment meet (possible gain of time, structure of treatment, influence of therapeutic relationship)
    - What is the most important aspect of this?
- What makes the blended treatment attractive to therapists?
- Which needs of the therapist does the blended treatment not meet?
- What do you need to make blended treatment part of your treatment (more time/technical support/training)?
- Does the blended treatment make you perform your work as a therapist differently than before?
- [Statement mobile voting system]: All therapists must provide the blended treatment (yes/no).

Profile of the therapist:
- What type of therapist do you have to be to be able or willing to treat blended?
- What type of therapist should you not be to be able or willing to treat blended?
- [Statement mobile voting system]: If you may decide, would you continue to use the blended treatment (yes/no)?
- What could possibly be done differently?

1.4. Facilitators and barriers
[Question mobile voting system]: Which implementation barrier is your number 1 (open answer)?
- What are the most important impeding factors for you as a therapist in implementing blended treatment in your daily work?
    - And what are the most important solutions for this?
- [Question mobile voting system]: Which implementation facilitator is your number 1 (open answer)?
- What are the most important facilitating factors for you as a therapist when implementing blended treatment in your daily work?

1.5. Satisfaction and usability
- [Question mobile voting system]: Patients are about the blended treatment ...(very dissatisfied/quite dissatisfied/quite satisfied/very satisfied).
- [Question mobile voting system]: Therapists are about the blended treatment ...(very dissatisfied/quite dissatisfied/quite satisfied/very satisfied).
- [Question mobile voting system]: Patients find the platform user-friendly (disagree entirely/disagree/neutral/I agree/strongly agree).
- [Question mobile voting system]: Therapists find the platform user-friendly (disagree entirely/disagree/neutral/I agree/strongly agree).

[Presentation by moderator: introduction of the results of the CSQ and SUS of both patients and practitioners. This concerns the meaning and value of satisfaction (CSQ) and user-friendliness/usability (SUS)]
- What do you think of the results regarding patient satisfaction?
- What do you think of the results regarding the satisfaction of the therapists?
- What do you think of the results regarding user-friendliness for patients?
- What do you think of the results regarding user-friendliness for the therapists?

File 2: Topic list semi-structured interviews experienced therapists

1. Introduction

This focus group is being held in the context of the MasterMind research. MasterMind stands for: MAnagement of mental health diSorders Through advancEd technology and seRvices-telehealth for the MIND. MasterMind is a large European study. It is an implementation study in which cohorts of patients are followed in 11 countries who receive Internet treatment for depression. Expectations are high and the results are promising, but Internet treatments are still used relatively little in practice. We therefore want to investigate how this treatment can best be applied, which factors are facilitating and which ones actually get in the way of implementation.

In the Netherlands, we are investigating how blended CBT treatment is applied in basic and specialist mental healthcare for patients with depression older than 18 years. Data is collected from 300 patients, practitioners, and the representatives of the mental healthcare organizations, including your organization.

With this interview we want to know wat your perspective, as a therapist, is on the implementation of Internet-based interventions in the treatment of depression.

1.1. Grand tour question:

What is your opinion about Internet-based interventions in the treatment of depression?

1.2. Barriers

Now, I would like to ask you about the barriers for using bCBT:

- What are the most important barriers for providing blended CBT for depression in routine practice?
- Why do you think that bCBT for depression isn't used often in routine practice?
  - Are there more aspects that prevent you (or other) from using bCBT?
  - Do you have negative experiences in using bCBT for depression?

1.3. Facilitators

Now, I would like to ask you about the facilitators for using bCBT:

- What are the most important facilitators providing blended CBT for depression in routine practice?
- What would help you (or others) as a therapist to treat more patients with a depression with bCBT?
  - Are there more aspects that motivate you (or others) to use bCBT?
  - Do you have positive experiences in using bCBT for depression?

1.4. Barriers and facilitators: therapist factors

Now, I would like to elaborate on the barriers and facilitators for using bCBT for depression in routine practice from the perspective of you as a therapist:

- How well do you know the different treatment elements of the online platform (e.g. platform, mobile application, treatment protocol)?
- What do you know about the effectiveness of bCBT?
- What kind of training is necessary for therapists to provide bCBT?
- What skills does a therapist need to treat patients with a depression with bCBT?
  - What do you think of your own skills?

- To what extent do you consider the use and integration of online elements in the treatment of depression as a part of your role as a therapist?
- Considering your experience, what would you add or remove from the bCBT?
- What is your opinion about the advantages and possibilities of bCBT?
    - What are the advantages/possibilities of bCBT vs regular CBT for you as a therapist?
    - What are the advantages/possibilities of bCBT vs regular CBT for your patients?
- What is your opinion about the disadvantages and risks of bCBT ?
    - What are the disadvantages/risks of bCBT vs regular CBT for you as a therapist?
    - What are the disadvantages/risks of bCBT vs regular CBT for your patients?
- What aspects of bCBT do you like most?
- Are you planning on providing bCBT for patients with depression in the future?
- What would help you as a therapist to provide bCBT more frequently in the future? Is this also applicable to your colleagues?
- To what extent can you imagine that the possibilities of bCBT will be expanded?
- Have your colleagues, patients or their family members your validation of bCBT reinforced? Or are you rather discouraged by them?
- To what extent do you feel that patients are willing to use bCBT?
- What should happen to make bCBT for depression a standard way of treatment?

[Added questions]
- What do you think is the desired proportion of online and face-to-face sessions? (e.g. 50–50, 80–20)
- What do you think is the desired way to 'blend' the online sessions in the treatment, from the beginning or at a later time point?
- What is your experience with writing online feedback?

1.5. Barriers and facilitators: patient factors
Now, on a patient level:
- For what kind of patients is bCBT eligible? And for who is it unsuitable?
- What are reasons to not use bCBT for a patient?
- How do you offer bCBT to patients?
- What are the reactions of patients when they are offered bCBT?
    - What reactions of patients keep you from not offering bCBT?
- Are there more or other barriers in the interaction with the patient or in the therapeutic relationship with bCBT?
- How is the therapeutic relationship developed in bCBT?

1.6. Barriers and facilitators: organizational factors
Now, on an organizational level:
- What existing resources, procedures, and work routines influence the use of bCBT in your organization? And how?
    - Which are facilitating and which form a barrier for implementation?
- What resources, procedures, and work routines would help to offer bCBT in a standardized way in routine practice?
- How can bCBT simplify the therapeutic and administrative work?

1.7. Barriers and facilitators: health insurance factors

Now, on a health insurance level:

- Are the (social-economical) factors from outside of the organization that hinders the implementation of bCBT?
- Are the (social-economical) factors from outside of the organization that facilitates the implementation of bCBT?
- What is the role of the health insurance companies in the implementation of bCBT?
- Which unmet need in the treatment of depression can be fulfilled with bCBT?

1.8. Closing question:

- Are there any issues that I didn't ask, but are important to consider in this study?

File 3: Topic list semi-structured interviews non-experienced therapists

1. Introduction

This focus group is being held in the context of the MasterMind research. MasterMind stands for: MAnagement of mental health diSorders Through advancEd technology and seRvices-telehealth for the MIND. MasterMind is a large European study. It is an implementation study in which cohorts of patients are followed in 11 countries who receive Internet treatment for depression. Expectations are high and the results are promising, but Internet treatments are still used relatively little in practice. We therefore want to investigate how this treatment can best be applied, which factors are facilitating and which ones actually get in the way of implementation.

In the Netherlands, we are investigating how blended CBT treatment is applied in basic and specialist mental healthcare for patients with depression older than 18 years. Data is collected from 300 patients, practitioners, and the representatives of the mental healthcare organizations, including your organization.

With this interview we want to know wat your perspective, as a therapist, is on the implementation of Internet-based interventions in the treatment of depression.

1.1. Grand tour question:

What is your opinion about Internet-based interventions in the treatment of depression?

1.2. Barriers

Now, I would like to ask you about the barriers for using bCBT:

- What are the most important barriers for providing blended CBT for depression in routine practice?
- Why do you think that bCBT for depression isn't used often in routine practice?
    - Are there more aspects that prevent you (or other) from using bCBT?

1.3. Facilitators

Now, I would like to ask you about the facilitators for using bCBT:

- What are the most important facilitators providing blended CBT for depression in routine practice?
- What would help you (or others) as a therapist to treat more patients with a depression with bCBT?
    - Are there more aspects that motivate you (or others) to use bCBT?

1.4. Barriers and facilitators: therapist factors

Now, I would like to elaborate on the barriers and facilitators for using bCBT for depression in routine practice from the perspective of you as a therapist:

- How well do you know the different treatment elements of the online platform (e.g. platform, mobile application, treatment protocol)?
- What do you know about the effectiveness of bCBT?
- What kind of training is necessary for therapists to provide bCBT?
- What skills does a therapist need to treat patients with a depression with bCBT?
    - What do you think of your own skills?
- What is your opinion about the advantages and possibilities of bCBT?
    - What are the advantages/possibilities of bCBT vs regular CBT for you as a therapist?
    - What are the advantages/possibilities of bCBT vs regular CBT for your patients?

- What is your opinion about the disadvantages and risks of bCBT ?
  - What are the disadvantages/risks of bCBT vs regular CBT for you as a therapist?
  - What are the disadvantages/risks of bCBT vs regular CBT for your patients?
- Are you planning on providing bCBT for patients with depression in the future?
- What would help you as a therapist to provide bCBT more frequently in the future? Is this also applicable to your colleagues?
- To what extent can you imagine that the possibilities of bCBT will be expanded?
- Have your colleagues, patients or their family members your validation of bCBT reinforced? Or are you rather discouraged by them?
- To what extent do you feel that patients are willing to use bCBT?
- What should happen to make bCBT for depression a standard way of treatment?

[Added questions]

- What do you think is the desired proportion of online and face-to-face sessions? (e.g. 50–50, 80–20)
- What do you think is the desired way to 'blend' the online sessions in the treatment, from the beginning or at a later time point?
- What is your experience with writing online feedback?

1.5. Barriers and facilitators: patient factors

Now, on a patient level:

- For what kind of patients is bCBT eligible? And for who is it unsuitable?
- What are reasons to not use bCBT for a patient?
- How would you offer bCBT to patients?
- Are there more or other barriers in the interaction with the patient or in the therapeutic relationship with bCBT?
- To what extent do you think that the therapeutic relationship is developed in bCBT?

1.6. Barriers and facilitators: organizational factors

Now, on an organizational level:

- What existing resources, procedures and work routines influence the use of bCBT in your organization? And how?
  - Which are facilitating and which form a barrier for implementation?
- What resources, procedures and work routines would help to offer bCBT in a standardized way in routine practice?
- How would bCBT simplify the therapeutic and administrative work?

1.7. Barriers and facilitators: health insurance factors

Now, on a health insurance level:

- Are the (social-economical) factors from outside of the organization that hinders the implementation of bCBT?
- Are the (social-economical) factors from outside of the organization that facilitates the implementation of bCBT?
- What is the role of the health insurance companies in the implementation of bCBT?
- Which unmet need in the treatment of depression can be fulfilled with bCBT?

1.8. Closing question:

Are there any issues that I didn't ask, but are important to consider in this study?
